# Attractant or Repellent? Behavioral Responses to Mammalian Blood Odor and to a Blood Odor Component in a Mesopredator, the Meerkat (*Suricata suricatta*)

**DOI:** 10.3389/fnbeh.2018.00152

**Published:** 2018-07-23

**Authors:** Henrik Pettersson, Mats Amundin, Matthias Laska

**Affiliations:** ^1^Department of Physics, Chemistry and Biology, Linköping University, Linköping, Sweden; ^2^Kolmården Wildlife Park, Kolmården, Sweden

**Keywords:** blood odor, epoxydecenal, behavior, mesopredator, meerkats, *Suricata suricatta*

## Abstract

It is well-established that the odor of mammalian blood is attractive to top predators such as tigers and wolves and aversive to prey species such as mice and rats. Recent studies have shown that the mammalian blood odor component *trans*-4,5-epoxy-(E)-2-decenal (TED) elicits corresponding behavioral responses in these two groups of mammals. Here we assess whether a mesopredator, that is, a small-bodied carnivorous mammal that is both predator and prey, is attracted to or repelled by the odor of mammalian blood and TED. To this end, we assessed the behavior of a group of 15 captive meerkats (*Suricata suricatta*) when presented with wooden logs that were impregnated either with horse blood or with TED, and compared it to their behavior toward a fruity odor (*iso*-pentyl acetate) and a near-odorless solvent (diethyl phthalate). We found that the meerkats displayed significantly more interactions with the odorized wooden logs such as sniffing and pawing when these were impregnated with the two prey-associated odors compared to the two non-prey-associated odors. Most importantly, no significant difference was found in the number of interactions with the wooden logs impregnated with horse blood and TED, respectively. These results demonstrate that meerkats, despite being small-bodied mesopredators, are clearly attracted to the odor of mammalian blood. Further, the results suggest that a single blood odor component can be as efficient as the odor of real blood in eliciting behavioral responses in this herpestid mammal, similar to previous findings in feline and canine top predators.

## Introduction

The vast majority of naturally occurring odor stimuli are highly complex mixtures composed of dozens or even hundreds of volatile compounds (Ohloff, 1994; Sell, 2014). Olfactory systems are therefore faced with the problem to recognize the identity of an odor stimulus despite permanent fluctuations in its composition or intensity due to changes in the odor source itself (e.g., ripening of a fruit) or the environment (e.g., convection of volatiles in air currents) (Riffell et al., 2009). How the olfactory system achieves and maintains stimulus identity of complex odor mixtures is still not fully understood (Thomas-Danguin et al., 2014).

One possible strategy for an animal to recognize a complex odor mixture is to rely on only one or a few “key” or “character impact” compound(s) which determine its odor identity (Dunkel et al., 2014). This strategy, of course, requires that the compound in question is reliably present in the odor mixture and that it can be reliably detected against the noise of the other compounds (Nehring et al., 2013). Thus, a high olfactory sensitivity for such a “key” or “character impact” compound should be expected in species for which the corresponding odor mixture is behaviorally relevant (Laska et al., 2005; Sarrafchi et al., 2013).

Behavioral tests assessing the ability of animals to recognize and to properly respond to the presentation of behaviorally relevant odors yielded rather mixed results concerning the efficiency of single compounds that are part of a complex odor mixture to elicit such adaptive behavioral responses. With regard to food odors, for example, recent studies found that frugivorous mammals do not seem to rely on single compounds to assess the degree of ripeness of a fruit but rather on the relative abundance of several compounds (Hodgkison et al., 2013; Nevo et al., 2015). Concerning the olfactory recognition of predators by prey species via body-borne odors, some studies found that single compounds are sufficient to elicit avoidance responses whereas other studies failed to find such effects or reported the behavioral responses to be weaker compared to those elicited by the full odor mixture (for a review, see Apfelbach et al., 2017).

The odor of blood has been shown to be attractive to mammalian top predators such as tigers (Nilsson et al., 2014) and wolves (Arshamian et al., 2017) and to be aversive to mammalian prey species such as the mouse (Sandnabba, 1997; Lahger and Laska, 2018) and the rat (Stevens and Saplikoski, 1973; Hornbuckle and Beal, 1974; Mackay-Sim and Laing, 1981). Interestingly, the tigers and wolves were equally attracted to and the mice were equally repelled by *trans*-4,5-epoxy-(E)-2-decenal (TED), a single component of mammalian blood odor which has been described by humans as having a typical “metallic, blood-like” odor quality (Buettner and Schieberle, 2001). These findings suggest that TED might indeed be a “key” or “character impact” compound which determines or at least contributes to the reliable recognition of blood odor. This notion is further supported by the finding that mice are extraordinarily sensitive to TED with olfactory detection thresholds in the ppt (parts per trillion) range (Sarrafchi and Laska, 2017).

Meerkats *(Suricata suricatta)* are carnivorous mammals belonging to the mongoose family (Herpestidae). They have well-developed olfactory brain structures (Gittleman, 1991; van Valkenburgh et al., 2014) and are known to strongly rely on olfactory cues in the context of social communication (Jordan, 2007; Mares et al., 2011; Leclaire et al., 2013). Similarly, meerkats have been reported to use their sense of smell for predator avoidance (Hollén and Manser, 2007; Zöttl et al., 2013) and for foraging and food selection (Leclaire, 2017). Due to their small body mass of 0.7–1.2 kg (van Staaden, 1994), they are typical mesopredators, meaning that they are both predators of smaller prey species and, at the same time, prey to larger predators. Their diet, although primarily based on arthropods, includes up to 20% (by volume) of small-bodied mammals and other vertebrates such as birds, eggs, lizards, and snakes (van Staaden, 1994; Doolan and Macdonald, 1996). Therefore, it should be interesting to assess whether meerkats are attracted to or repelled by the odor of blood and if they also display the same behavior toward TED.

It was therefore the aim of the present study to (1) assess behavioral responses of meerkats to mammalian blood odor and to the mammalian blood odor component *trans*-4,5-epoxy-(E)-2-decenal, (2) to compare their behavioral responses to those toward a fruity odor and a near-odorless control, and (3) to compare their behavioral responses to those of top predators and prey species tested previously on the same odor stimuli.

## Materials and Methods

### Ethics Statement

The experiments reported here comply with the *Guide for the Care and Use of Laboratory Animals* (8th edition, National Research Council, 2011) and also with current Swedish laws. They were performed according to a protocol approved by the ethical board of the Swedish Board of Agriculture (Jordbruksverket, protocol # 31-2647/10).

### Animals

The study was conducted at Kolmården Wildlife Park, near Norrköping, Sweden. A group of 15 meerkats (*S. suricatta*), comprising twelve males and three females ranging from a few months to 10 years of age, was observed. All animals were born in captivity. The enclosure of the meerkats was composed of an indoor and an outdoor part, connected by a sliding door. The indoor enclosure was 40 m^2^ and had a ground substrate of sand. It contained standing brush material, tree stumps of a varied height, and different hiding places such as wooden nest boxes. The outdoor enclosure was 330 m^2^ with mainly earth as ground substrate. In addition to tree stumps, the outdoor enclosure also had a grassy area, coniferous trees, rocks and bushes scattered throughout the area. The ground substrate of both the indoor and the outdoor enclosure allowed the animals to dig burrows and tunnels. The meerkats could freely choose between the indoor and the outdoor enclosure during the daytime, but were kept indoors over night. They were provided with food three times per day (in the morning, that is, about 60 min prior to the start of the day’s observations; around noon, that is, during the hour between the morning and the afternoon observations; and in the afternoon, that is, after the end of the day’s observations). Their food consisted of mice, baby chicken, pieces of meat from different even-toed ungulate species (such as deer and antelope, but not from odd-toed ungulate species such as horses), cat food pellets (Four Friends Senior, Västerås, Sweden), fruit (banana, apple, different berries), crickets, mealworms, chicken eggs.

### Odor Stimuli

The four odor stimuli used were:

Blood from a domestic horse (*Equus ferus caballus*). The blood was collected directly after the horse was euthanized and immediately deep-frozen in aliquots of 0.5 ml at −20°C. On the morning of each testing day, five aliquots of horse blood were thawed and warmed up to approximately 25°C.

*trans*-4,5-epoxy-(E)-2-decenal (CAS# 134454-31-2), henceforth abbreviated as TED. This odorant has been identified as a volatile component in mammalian blood and evokes a typical “metallic, blood-like” odor quality in humans (Buettner and Schieberle, 2001). It was presented at a dilution of 1:100 (in diethyl phthalate) from a stock solution of 5 mg/ml.

The rationale for using these two odor stimuli was that horse blood and *trans*-4,5-epoxy-(E)-2-decenal had also been used in previous studies assessing behavioral responses of top predators (Nilsson et al., 2014; Arshamian et al., 2017) and a prey species (Lahger and Laska, 2018) to blood odor.

*iso*-pentyl acetate (CAS# 123-92-2). This odorant has been identified as a volatile component in a variety of fruits and evokes a typical “banana-like” odor quality in humans (Burdock, 2009). It was presented at a dilution of 1:1,000 (in diethyl phthalate).

Diethyl phthalate (CAS# 84-66-2). This organic solvent is near-odorless and was used both for diluting the two monomolecular odorants mentioned above and as a blank stimulus.

The concentrations mentioned above for the blood odor component TED and for the fruity odor (*iso*-pentyl acetate) were chosen in order to provide stimuli that were clearly detectable for humans, but not overwhelmingly strong so that a relatively close contact to the odor source was necessary to detect them.

### Experimental Procedure

The four different odor stimuli were presented to the animals using wooden (spruce) logs of 48 cm × 4.5 cm × 4.5 cm. Each log was impregnated with 0.5 ml of a given odor stimulus immediately prior to each presentation. The odor stimulus was applied on the two largest surfaces of a log using a micropipette and then spread over the surface using a paint brush. Plastic gloves were used whenever the logs were handled to avoid that they were contaminated with human odor. Five logs, impregnated with the same odor stimulus, were used during a given experimental day.

At the start of each experimental day, five freshly odorized wooden logs were placed into the outdoor enclosure of the meerkats. Care was taken to present the wooden logs to the animals at positions that differed between experimental days and, at the same time, allowed the experimenter to see all five logs at least at the start of the observation. Immediately after the logs were put in place, the animals were allowed access into the outdoor enclosure and were observed for 3 h in the morning and 3 h in the afternoon (between 08:00 a.m. and 04:00 p.m.). At the end of each experimental day the wooden logs were removed from the enclosure. Experiments were only performed on non-rainy days to prevent the odor stimuli from being washed away by the rain. At least 1 day was interspersed between consecutive presentations of odorized wooden logs. Each of the four odor stimuli was presented for a total of five times in a pseudo-randomized order which resulted in a total of 20 experimental days.

**FIGURE 1:**
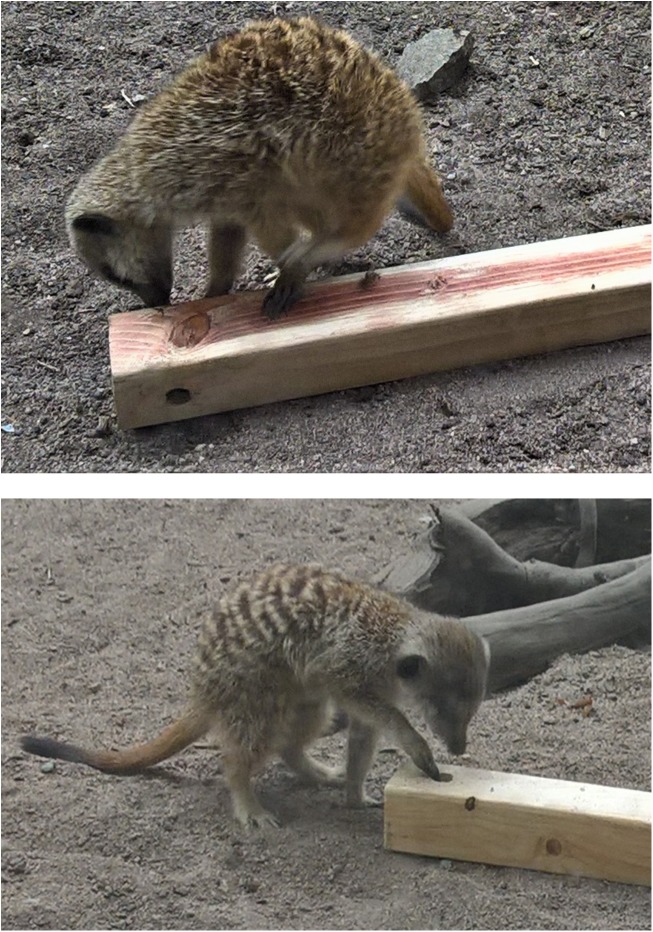
The two behaviors performed most often by the meerkats. **(Upper)** A meerkat sniffing at an odorized wooden log. **(Lower)** A meerkat pawing at an odorized wooden log.

Continuous sampling was used to record the occurrence of each interaction with a wooden log which was visible to the experimenter. A total of twelve behaviors involving different kinds of interaction with or immediate behavioral responses to the inspection of an odorized wooden log were considered (**Table 1** and **Figure 1**). During one experimental day per odor stimulus, the duration of the behaviors was also recorded using a stopwatch.

**Table 1:** Ethogram of all behaviors considered in the present study.

Functional term	Description
Sniffing	Using the nose to investigate a wooden log
Pawing	Using the paw or claws to scratch a wooden log
Licking	Using the tongue to investigate a wooden log
Biting	Using the teeth to investigate a wooden log
Toying	Moving or otherwise manipulating a wooden log
Flehmen	Curling of the upper lip and “grimacing” when investigating a wooden log
Self-impregnating	Rubbing the face or other body part at a wooden log
Scent-marking	Depositing body-borne odors onto a wooden log
Orientating	Turning head, ears, or eyes following an interaction with a wooden log
Guarding	Resting close to or on top of a wooden log
Vocalizing	Producing sounds during or following an interaction with a wooden log
Fleeing	Rapidly moving away following an interaction with a wooden log

### Data Analysis

Differences in the frequency of occurrence of behavioral responses between odor stimuli were assessed using the Chi-square test. Within-species comparisons of the duration of behavioral responses were performed using the Friedman test. Correlational analyses were performed by calculating Spearman rank-order coefficients *r*_s_ which were tested for significance by computing *z*-scores. As the number of animals differed between the species that were compared, we calculated the number of interactions *per animal* by dividing the total number of interactions observed in a given species by the number of animals. All statistics were performed using SPSS, version 22.0.

## Results

### Number of Interactions

Across all 20 observation days with the four different odor stimuli combined the meerkats interacted in total 2850 times with the odorized wooden logs. Ten out of the twelve behaviors described in the ethogram were observed (**Table 2**). Sniffing was by far the most frequently displayed behavior with 1970 interactions, representing 69% of all observations. Pawing was another frequently observed behavior with 834 occurrences, accounting for 29% of of all observations. Thus, sniffing and pawing combined accounted for >98% of all interactions with the odorized wooden logs that the meerkats displayed. Licking and flehmen were never displayed by the meerkats with any of the four odor stimuli.

**Table 2:** Number of interactions with the odorized wooden logs in the meerkats (*n* = 15).

Behavior	Horse blood	Blood component	Fruity odor	Blank control	Total
Sniffing	598	551	378	443	1970
Pawing	252	258	126	198	834
Licking	0	0	0	0	0
Biting	0	2	2	3	7
Toying	1	6	0	0	7
Flehmen	0	0	0	0	0
Self-impregnating	0	1	1	0	2
Scent-marking	0	0	0	4	4
Orientating	4	2	7	3	16
Guarding	1	0	2	1	4
Vocalizing	0	1	0	0	1
Fleeing	0	4	1	0	5
**Total**	**856**	**825**	**517**	**652**	**2850**

### Comparison Between Odor Stimuli

A comparison between the four odor stimuli showed that the meerkats displayed a significantly higher number of interactions with the wooden logs when these were odorized with horse blood compared to when they were odorized with the fruity odor (χ^2^ = 83.701, *P* < 0.0001) and the blank control (χ^2^ = 27.597, *P* < 0.0001). Similarly, the meerkats interacted significantly more often with the wooden logs when these were odorized with the blood odor component TED compared to the the fruity odor (χ^2^ = 70.689, *P* < 0.0001) and the blank control (χ^2^ = 20.263, *P* < 0.0001). In contrast, the number of interactions with the wooden logs did not differ significantly between the horse blood and the blood odor component TED (χ^2^ = 0.572, *P* = 0.450).

### Duration of Interactions

The mean duration of interactions with the odorized wooden logs was 3.1 ± 5.5 s for the four different odor stimuli combined (**Table 3**).

**Table 3:** Duration of interactions with the odorized wooden logs in the meerkats (*n* = 15).

Horse blood	Blood component	Fruity odor	Blank control	Total
2.2 ± 1.9	3.1 ± 6.3	4.5 ± 8.0	2.7 ± 4.0	3.1 ± 5.5

*Given are mean (±SD) values in seconds*.

No significant differences in the mean duration of interactions with the wooden logs were found between any of the four odor stimuli (Friedman ANOVA: χ = 6.01, *P* = 0.111).

### Variability Between Sessions

**FIGURE 2:**
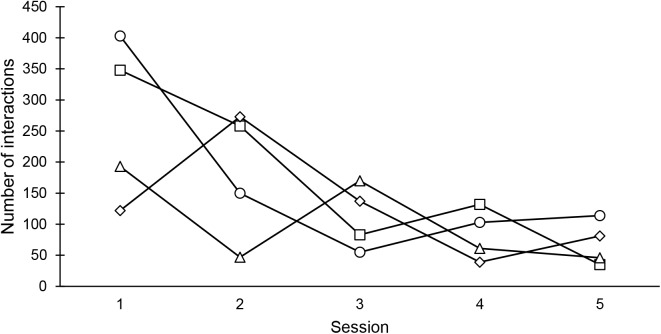
The number of interactions with the odorized logs across the five sessions performed per odor stimulus. circles: blood odor component TED [*trans*-4,5-epoxy-(E)-2-decenal]; squares: horse blood; triangle: fruity odor (*iso*-pentyl acetate); diamond: blank control (diethyl phthalate).

No significant correlation between the number of interactions with the wooden logs across the five sessions was found with any of the four odor stimuli (Spearman test, horse blood: *r*_s_ = −0.70, *P* > 0.05; blood odor component: *r*_s_ = −0.60, *P* > 0.05; fruity odor: *r*_s_ = −0.70, *P* > 0.05; solvent: *r*_s_ = −0.60, *P* > 0.05). However, with all four odor stimuli the number of interactions with the wooden logs was higher in the first compared to the fifth session (**Figure 2**). Accordingly, a non-significant trend for a decrease in the animals’ interest in the wooden logs across sessions was found as indicated by the negative correlation coefficients.

## Discussion

The results of the present study demonstrate that meerkats, small-bodied mesopredators, are clearly attracted to, and not repelled by, the odor of blood. Further, they show that the blood odor component *trans*-4,5-epoxy-(E)-2-decenal is as efficient in eliciting behavioral attraction responses in *S. suricatta* as the odor of real blood.

### Comparison Between Species

Our finding that the meerkats were clearly attracted to the odor of blood and to the blood odor component TED is not trivial considering that they are small-bodied mammals which are prey to a variety of larger-bodied predators (van Staaden, 1994). Thus, it should not have been surprising if the meerkats in our study would have behaved like mice and rats, that is, like a prey species and accordingly would have avoided the odor of blood (Hornbuckle and Beal, 1974; Lahger and Laska, 2018). If we further consider that the diet of meerkats is not exclusively based on smaller vertebrates but also includes a variety of non-vertebrate food items such as arthropods and plant material (van Staaden, 1994), it is actually somewhat unexpected that the meerkats behaved like top predators such as tigers and wolves (Nilsson et al., 2014; Arshamian et al., 2017) and were attracted to blood odor. This, in turn, suggests that the attraction to blood odor displayed by carnivorous mammals of the feline and canine families may be an evolutionarily old trait as the mongoose family (Herpestidae) to which the meerkats belong split from the other carnivore families about 25 million years ago (Nyakatura and Bininda-Emonds, 2012).

A comparison of the behavioral responses of the meerkats in the present study to those of several species of top predators tested previously with the same method and odor stimuli (Nilsson et al., 2014; Arshamian et al., 2017) shows that the meerkats displayed the highest number of interactions *per animal* with the odorized wooden logs (**Table 4**). This was true both when considering all four odor stimuli combined and separately.

**Table 4:** Number of interactions per animal with the odorized wooden logs.

Species	Horse blood	Blood component	Fruity odor	Blank control	Total
Meerkats^1^	57.1	55.0	34.5	43.5	190.0
Eurasian wolves^2^	48.1	45.0	17.6	14.4	125.1
African wild dogs^3^	11.8	23.6	10.6	7.9	53.9
Asian wild dogs^3^	13.4	14.2	6.3	4.3	38.2
South American Bush dogs^3^	54.0	52.1	29.7	22.4	158.2
Siberian tigers^3^	27.7	27.2	5.7	4.8	65.3

*(Data from ^1^present study, ^2^Arshamian et al., 2017, ^3^Nilsson et al., 2014).*

The total number of interactions with the odorized wooden logs *per animal* displayed by the meerkats was almost five times higher than that of the Asian wild dogs, and more than three times higher compared to the African wild dogs and Siberian tigers, respectively. The South American bush dogs and the Eurasian wolves displayed a higher interest *per animal* to all four odor stimuli than the other two top predators, but still lower numbers compared to the meerkats. This raises the question as to possible reasons underlying these between-species differences in their degree of interest toward the odorized wooden logs. Several possible explanations should be considered:

Although the possibility that differences in enclosure size might have affected the likelihood of an animal to encounter and interact with the odorized wooden logs cannot be ruled out completely, it seems unlikely to explain the above-mentioned between-species differences in interest as correlations between enclosure size (m^2^/animal) and interest in the odorized wooden logs (interactions/animal) were not significant for any of the four odor stimuli considered separately (Spearman test: real blood odor: *r*_s_ = −0.49, *p* = 0.33; TED: *r*_s_ = −0.43, *p* = 0.40; fruity odor: *r*_s_ = −0.60, *p* = 0.21; solvent: *r*_s_ = −0.49, *p* = 0.33) or combined (*r*_s_ = −0.43, *p* = 0.40). Similarly, although subadult mammals tend to be more reactive to novel objects and odor stimuli than adult conspecifics (Glickman and Sroges, 1966), differences in the age composition of the studied carnivore species are also unlikely to explain the observed between-species differences in interest toward the odorized wooden logs as with all six species under consideration >90% of all individuals were adults. Whether between-species differences in overall activity level may account for the finding that the meerkats displayed the highest interest in the odor stimuli used here needs further investigation. Although we did not systematically record the overall activity of the meerkats across the 6 h of observation per experimental day, it was obvious that they, similar to the other carnivore species tested in previous studies, alternated between phases of rest and phases of activity. Thus, there were at least no obvious between-species differences in this parameter.

Whether the observed between-species differences in interest toward the odorized wooden logs might reflect generic differences in their use of the sense of smell for exploring objects and/or odors is hard to decide. All six carnivore species are known to possess well-developed olfactory brain structures (Gittleman, 1991; van Valkenburgh et al., 2014) and all have been reported to use olfactory cues for social communication and foraging (Nowak, 2005). However, this does not exclude the possibility that the importance and use of the sense of smell may indeed differ among these carnivores. Thus, at this point no conclusive answer can be given as to why the meerkats of the present study displayed a markedly higher interest toward all four odor stimuli compared to the other carnivore species.

### Recognition of the Odor Identity of a Complex Odor Mixture

With regard to the reliable recognition of behaviorally relevant odors which are almost always complex mixtures of volatiles the olfactory system can adopt two different strategies:

The first strategy implies that the olfactory system relies on only one or a few “key” or “character impact” compounds which are part of a complex mixture and are used for the recognition of an odor. Accordingly, this strategy requires olfactory receptors that are highly specific to a given ligand and central circuits which allow for a quick and hard-wired translation of a chemical stimulus into a specific behavioral response (Wilson and Stevenson, 2006). Further, this strategy requires that the “key” or “character impact” compound in question is reliably present in the odor mixture and that it can be reliably detected against the noise of the other compounds that are part of the odor mixture as well as against the chemical background noise in the environment (Nehring et al., 2013). Thus, this strategy also requires a high olfactory sensitivity for such a compound in species for which the corresponding odor is behaviorally relevant.

The second strategy implies that the olfactory system relies on either the full bouquet of volatiles that comprise an odor mixture or at least on a larger proportion of its components for the recognition of an odor. Accordingly, this strategy does not require olfactory receptors to be highly specific but instead requires central circuits to create a unitary percept or “odor object” from a complex mixture of volatiles which is stable against fluctuations in the composition and intensity of the mixture components (Wilson and Stevenson, 2006). Thus, this strategy requires the olfactory system to generate patterns of activation which allow for fine discrimination of similar odor mixtures and a cut-off criterion which allows for distinguishing the odor object in question from similar odor objects. An example for the first strategy, in which the olfactory system relies on “key” or “character impact” compounds for odor recognition, would be the perception of certain pheromones, e.g., the rabbit mammary pheromone 2-methylbut-2-enal which has been identified as a component of rabbit milk and elicits a hard-wired behavioral response in rabbit pups (Coureaud et al., 2003; Schaal et al., 2003). This strategy is often connected to innate behavioral responses. An example for the second strategy, in which the olfactory system relies on a complex mixture of volatiles for odor recognition, would be the perception of individual body odors (Beauchamp and Yamazaki, 2003). This strategy is often connected to learned behavioral responses.

To which of these two strategies do our findings of the present study as well as those of previous studies on behavioral responses to blood odor and to the blood odor component *trans*-4,5-epoxy-(E)-2-decenal fit?

Our finding that the blood odor component TED elicited the same high degree of interest in the meerkats as the odor of real blood suggests that this monomolecular compound may serve as a “key” or “character impact” compound for the odor of blood in this and other carnivore species. However, we would like to emphasize that we do not know whether TED is “the” or just “a” component of blood odor that induces a behavioral attraction response. Considering the high number of volatiles that comprise the odor of blood (Kusano et al., 2013) it is not feasible to test all of them with a given species of animal. However, gas chromatography-olfactometry showed that TED was the only blood odor component described as having the typical “metallic, blood-like” quality that is characteristic for the odor of blood as perceived by humans (Rachamadugu, 2012).

*Trans*-4,5-epoxy-(E)-2-decenal is a product of lipid peroxidation (Buettner and Schieberle, 2001). Considering that this biochemical process is ubiquitous in the metabolism of mammals, it is very likely that TED is present in the blood odor of all mammals. Thus TED is likely to fulfill the prerequisite of the first strategy mentioned above of being reliably present in the odor mixture. Unfortunately, the olfactory detection threshold for TED is not known for any of the predator species that are attracted by this blood odor component. However, human subjects and mice which are both repelled by the odor of TED (Arshamian et al., 2017; Lahger and Laska, 2018) have been shown to be extraordinarily sensitive to this compound with threshold values in the ppt (parts per trillion) range (Buettner and Schieberle, 2001; Sarrafchi and Laska, 2017). Thus, there is at least circumstantial evidence that TED may fulfill the prerequisite of a “key” or “character impact” compound of being detectable at low concentrations.

To the best of our knowledge, no olfactory receptor has been identified so far as having TED as its specific ligand in any species. Thus, we do not know yet if TED may fulfill the prerequisite of the first strategy mentioned above of having an olfactory receptor that is highly specific to this ligand. Future studies should therefore aim at testing the specificity and sensitivity of olfactory receptors for TED.

Finally, we do not know whether the attraction response to both blood odor and TED observed in mammalian predators is innate, which would support the notion that the first strategy mentioned above might apply, or whether the attraction response is acquired. Thus, future studies should assess behavioral responses of newborn predators to these odors. The finding that laboratory-born mice that were naïve with regard to the odors of blood and TED clearly avoided both odors (Lahger and Laska, 2018) suggests that behavioral responses to TED might indeed be innate rather than acquired.

### The Bipolar Behavioral Effect of Blood Odor and TED

Most of the behaviorally relevant odors studied so far have one thing in common: they elicit a certain behavioral response in a given species, or in a given group of species, and they fail to elicit any response in other species, or other groups of species. It is a hallmark of pheromones, for example, to be species-specific and thus to elicit adaptive behavioral responses, e.g., mating or aggregation, in conspecifics but not in heterospecifics (Wyatt, 2014). Similarly, food odors usually elicit adaptive behavioral responses, e.g., foraging for or inspection of a food item, in those species that feed on a given type of food but are usually ignored by species that do not include this type of food into their diet (Stoddart, 1980). Predators are attracted by the odor of their prey whereas non-predators are usually indifferent to the odor of the prey species in question (Conover, 2007). Accordingly, prey species are repelled by the odor of their natural predator whereas non-prey species usually are not (Parsons et al., 2017).

The odors of blood and of its component *trans*-4,5-epoxy-(E)-2-decenal appear to be rather unique in the sense that they elicit a bipolar behavioral effect: an attraction response in predators (Nilsson et al., 2014; Arshamian et al., 2017) and an avoidance response in prey species (Hornbuckle and Beal, 1974; Lahger and Laska, 2018). In this context it is interesting to note that the avoidance response to both blood odor and TED displayed by mice suggests that these odors are likely to contain a warning cue rather than a fear cue as the animals significantly avoided these odor stimuli without showing behavioral indicators of fear such as freezing or defecation (Lahger and Laska, 2018). Similar behavioral responses indicative of a warning cue, but not a fear cue, have been reported in rats when presented with the odor of sick conspecifics (Arakawa et al., 2010). Future studies should therefore aim at elucidating the neural basis of these opposing behavioral responses, for example whether there are “labeled line”-like neural pathways mediating the connection between the perception of blood odor or of the blood odor component TED and behavioral attraction or avoidance responses.

## Author Contributions

HP, MA, and ML conceived the study, analyzed the data, and wrote the manuscript. HP collected the data. All authors critically revised the manuscript, approved the final version, and agree to be accountable for its content.

## Conflict of Interest Statement

The authors declare that the research was conducted in the absence of any commercial or financial relationships that could be construed as a potential conflict of interest.
